# Role of cytokines in immunomodulation during malaria clearance

**DOI:** 10.1097/MS9.0000000000002019

**Published:** 2024-04-03

**Authors:** Emmanuel Ifeanyi Obeagu

**Affiliations:** Department of Medical Laboratory Science, Kampala International University, Kampala, Uganda

**Keywords:** chemokines, cytokines, immunomodulation, inflammation, interleukin, malaria

## Abstract

Malaria remains a significant global health challenge, demanding a deeper understanding of host immune responses for effective clearance of the parasitic infection. Cytokines, as crucial mediators of the immune system, orchestrate a complex interplay during the various stages of malaria infection. Throughout the course of the disease, an intricate balance of pro-inflammatory and anti-inflammatory cytokines dictate the immune response’s outcome, influencing parasitic clearance and disease severity. During the initial stages, interleukins such as interleukin-12 (IL-12), interferon-gamma (IFN-γ), and tumour necrosis factor-alpha (TNF-α) play pivotal roles in activating innate immune cells, initiating the anti-parasitic response. Simultaneously, regulatory cytokines like interleukin-10 (IL-10) and transforming growth factor-beta (TGF-β) modulate this immune activation, preventing excessive inflammation and tissue damage. As the infection progresses, a delicate shift occurs, characterized by a transition to adaptive immunity, guided by cytokines like interleukin-4 (IL-4), interleukin-5 (IL-5), and interleukin-13 (IL-13), promoting antibody production and T-cell responses. Notably, the resolution of malaria infection crucially relies on a fine-tuned balance of cytokine networks. Dysregulation or imbalances in these mediators often result in immune hyperactivation, contributing to severe manifestations and prolonged infection. Understanding the multi-faceted roles of cytokines in malaria clearance offers promising avenues for therapeutic interventions. Targeting cytokine pathways to restore immune equilibrium or bolster protective responses could potentially enhance treatment strategies and vaccine development. In conclusion, the pivotal role of cytokines in immunomodulation during malaria clearance underscores their significance as potential targets for therapeutic interventions, offering promising prospects in the global fight against this infectious disease.

## Introduction

Highlights

- Malaria.
- Malaria and inflammation.
- Malaria and immunity.
- Cytokines in the pathogenesis of malaria.
- Mechanisms by which cytokines contribute to malaria clearance.
- Factors that influence the balance of pro-inflammatory and anti-inflammatory cytokines during malaria infection.
- Cytokine profile differences between individuals with different clinical manifestations of malaria, such as uncomplicated versus severe disease.
- Cytokine signatures associated with malaria severity.
- Immunomodulation in malaria
- Cytokine responses to *Plasmodium falciparum.*
- Cytokines and chemokines in malaria
- Outcomes and challenges to potential therapeutic interventions targeting cytokine pathways that have been explored in preclinical and clinical studies.
- Future directions.

Malaria,^1^. The complex interactions between the parasite and the host immune system play a crucial role in determining disease severity, clearance of the infection, and the development of protective immunity^2^. Among the key orchestrators of these immune responses are cytokines – small signalling molecules that regulate and modulate the immune system’s function^3^. The intricate balance between pro-inflammatory and anti-inflammatory cytokines serves as a critical determinant in the immune response against malaria^4^. Upon infection, the host’s innate immune system is activated, initiating a cascade of cytokine-mediated responses^5^. Pro-inflammatory cytokines such as interleukin-12 (IL-12), interferon-gamma (IFN-γ), and tumour necrosis factor-alpha (TNF-α) are among the early responders, crucial in activating macrophages and natural killer cells to combat the invading parasites^6^. Malaria remains a significant public health challenge, particularly in sub-Saharan Africa, where the majority of global cases and deaths occur. Certain countries in Asia and the Americas also have ongoing malaria transmission^6^.

Simultaneously, regulatory cytokines, notably interleukin-10 (IL-10) and transforming growth factor-beta (TGF-β), act as modulators, preventing excessive inflammation and immune-mediated tissue damage. This delicate balance ensures effective parasite clearance while limiting host-induced pathology^6–11^. As the infection progresses, the adaptive immune response comes into play, marked by the activation of T cells and the production of antibodies^12^. Cytokines such as interleukin-4 (IL-4), interleukin-5 (IL-5), and interleukin-13 (IL-13) drive this transition to adaptive immunity, facilitating the generation of specific antibodies against the parasite and the development of T-cell-mediated responses crucial for long-term immunity^13–16^.

However, dysregulation or imbalances in cytokine production can lead to immune hyperactivation, contributing to the severity of malaria symptoms^17^. Severe malaria, often associated with excessive cytokine responses, endothelial dysfunction, and systemic inflammation, highlights the critical role of cytokines in influencing disease outcomes^4^. Understanding the dynamic interplay of cytokines in malaria clearance is paramount for developing effective interventions. Targeting specific cytokine pathways presents potential opportunities for therapeutic strategies and vaccine development aimed at bolstering protective immune responses while mitigating immunopathology. In this context, this paper aims to comprehensively explore the intricate roles played by cytokines in immunomodulation during malaria clearance, shedding light on their significance as key regulators of host-parasite interaction and potential targets for malaria therapeutics.

## Aim

The aim of this review is to comprehensively examine and synthesize the current literature on the role of cytokines in immunomodulation during the clearance of malaria.

## Malaria

Malaria is a life-threatening infectious disease caused by Plasmodium parasites transmitted to humans through the bites of infected female Anopheles mosquitoes. There are several species of Plasmodium parasites, with *Plasmodium falciparum* and *Plasmodium vivax* being the most common and dangerous to humans^18–22^. Upon entering the bloodstream through a mosquito bite, the parasites travel to the liver, where they multiply^23^. After a period of incubation, they re-enter the bloodstream and infect red blood cells, leading to the characteristic symptoms of malaria^24^. These symptoms include fever, chills, sweats, headaches, nausea, and body aches. In severe cases, malaria can lead to complications such as anaemia, cerebral malaria (affecting the brain), respiratory distress, and organ failure, which can be fatal if not treated promptly and effectively^25^.

Malaria remains a significant global health challenge, particularly affecting tropical and subtropical regions where the Anopheles mosquito thrives. Factors contributing to its persistence include drug resistance, insecticide resistance in mosquitoes, socio-economic conditions, and challenges in accessing healthcare and preventive measures in affected regions^26^. Preventive measures against malaria include the use of insecticide-treated bed nets, indoor residual spraying to control mosquitoes, chemoprevention strategies in high-risk populations, and anti-malarial drugs^27^. Diagnosis and prompt treatment with effective anti-malarial medications are crucial to preventing severe illness and death from malaria^28^. Efforts to control and eliminate malaria involve a multi-faceted approach, including the development of new drugs, vaccines, vector control strategies, and improved access to healthcare services in affected areas^29^. Global initiatives and collaborations aim to reduce the burden of malaria and work towards the ultimate goal of eradicating this disease^30^.

## Malaria and inflammation

Malaria infection triggers a complex interplay between the parasite, the immune system, and inflammation^31^. Inflammation, a fundamental part of the immune response, plays a dual role in malaria: it contributes to the control and elimination of the parasite while also being a major factor in the pathogenesis of the disease^32^. When Plasmodium parasites infect red blood cells and multiply within them, they induce the release of various molecules, including hemozoin and parasite-derived factors, which stimulate the host’s immune cells to produce pro-inflammatory cytokines such as tumour necrosis factor-alpha (TNF-α), interleukin-1β (IL-1β), and interleukin-6 (IL-6). These cytokines, along with other mediators, trigger an inflammatory response aimed at controlling the infection^33^. In the initial stages of malaria infection, the pro-inflammatory response contributes to parasite clearance by activating immune cells like macrophages to phagocytose-infected red blood cells and by enhancing the killing of parasites^33^.

However, excessive or dysregulated inflammation can lead to immunopathology and severe manifestations of malaria^34^. High levels of pro-inflammatory cytokines can contribute to endothelial dysfunction, coagulopathy, and organ damage^35^. For instance, in cerebral malaria, increased inflammation and the sequestration of infected red blood cells in the brain’s blood vessels can lead to neurological complications and even death^36^. Additionally, the balance between pro-inflammatory and anti-inflammatory responses is crucial. Anti-inflammatory cytokines, such as IL-10, help regulate the immune response and prevent excessive inflammation. However, an overproduction of anti-inflammatory cytokines may impair the immune system’s ability to effectively clear the parasite^31,37,38^. Understanding the intricate relationship between malaria and inflammation is vital for developing targeted therapies that modulate the immune response to achieve parasite clearance while minimizing immunopathology. Research into immunomodulatory strategies and therapies that balance the inflammatory response during malaria infection remains an important area of study for improving treatment outcomes and reducing the burden of severe malaria.

## Malaria and immunity

Malaria and immunity have a complex relationship influenced by various factors, including the Plasmodium parasite’s ability to evade the immune system and the host’s immune response to the infection^39^. The immune response to malaria involves both innate and adaptive immunity. Initially, the innate immune system responds to the presence of the parasite through mechanisms such as inflammation, phagocytosis, and the release of cytokines (small signalling molecules) by immune cells. This early response aims to limit the spread of the parasite^40^. Adaptive immunity, particularly cell-mediated and humoral immune responses, plays a crucial role in controlling and clearing the infection^41^. T cells and B cells, part of the adaptive immune system, produce specific antibodies and target infected cells to eliminate the parasite^42^. Individuals living in malaria-endemic regions gradually develop some level of immunity through repeated exposure to the parasite, resulting in a partially protective immune response that can mitigate the severity of subsequent infections^43^.

However, Plasmodium parasites have evolved various strategies to evade the immune system, such as changing their surface antigens, which allows them to evade detection by antibodies and immune cells^44^. This ability to evade the immune response contributes to the parasite’s persistence and the recurrence of malaria infections in individuals living in endemic areas^45^. Immunity to malaria is not absolute and can wane over time, particularly in the absence of continuous exposure to the parasite. This leads to the susceptibility of individuals, including travellers or those living in non-endemic regions, to severe forms of malaria upon re-exposure^46^. Factors influencing immunity to malaria include the individual’s age, genetic factors, the strain of the parasite, and the frequency and intensity of exposure. Pregnant women and young children, especially in endemic areas, are particularly vulnerable to severe forms of the disease due to their less-developed or reduced immunity^47,48^.

## Cytokines in the pathogenesis of malaria

Malaria, caused by Plasmodium parasites, presents a complex interplay between the invading pathogen and the host immune system. Cytokines, as key mediators of immune responses, orchestrate a dynamic and intricate network of signalling pathways that significantly influence the pathogenesis and progression of the disease^49,50^. The early stages of malaria infection trigger the activation of innate immune cells, eliciting a rapid cytokine response. Pro-inflammatory cytokines such as IL-12, IFN-γ, and TNF-α play pivotal roles in initiating and amplifying the immune response against the parasites. These cytokines activate macrophages, natural killer cells, and other effector cells to limit initial parasite growth^51–53^.

Conversely, regulatory cytokines, including IL-10 and TGF-β, function as crucial modulators to prevent excessive inflammation and immunopathology. They regulate the intensity of the immune response, thus balancing the protective immunity against the parasites with the potential for immune-mediated tissue damage^11^. The delicate equilibrium between pro-inflammatory and anti-inflammatory cytokines throughout the course of infection profoundly impacts disease severity and outcome. Dysregulation or imbalances in cytokine production can lead to immune hyperactivation, contributing to the development of severe malaria. This phenomenon is often characterized by systemic inflammation, endothelial dysfunction, and multi-organ complications^54,55^. Furthermore, the specific profile of cytokines varies depending on the Plasmodium species involved, with different species evoking distinct cytokine responses. For instance, *Plasmodium falciparum* infections often trigger more pronounced pro-inflammatory cytokine responses, whereas *Plasmodium vivax* infections may induce a different cytokine profile^56,57^.

Understanding the nuanced roles of cytokines in malaria pathogenesis provides valuable insights into disease mechanisms and guides the development of novel therapeutic interventions. Strategies aimed at modulating cytokine responses to restore immune balance while maintaining protective immunity hold promise in improving treatment outcomes and reducing malaria-associated morbidity and mortality. Cytokines stand at the forefront of malaria pathogenesis, intricately regulating the immune response and influencing disease severity. Further exploration of cytokine dynamics and their interplay with the host immune system is essential for developing targeted interventions and advancing malaria control strategies^4^. Table 1: Pro-inflammatory Cytokines, Table 2 shows anti-inflammatory cytokines, Table 3 shows chemotactic cytokines (chemokines) and Table 4 shows Th17-related cytokines^4^.

**Table 1:** Pro-inflammatory cytokines

Cytokine	Source	Function during malaria clearance
TNF-α	Macrophages, T cells	Activates immune cells, promotes inflammation, and induces fever.
IL-1β	Macrophages, dendritic cells	Enhances inflammation and contributes to the activation of adaptive immune responses.
IL-6	Macrophages, T cells	Stimulates the acute-phase response and supports the differentiation of immune cells.
IFN-γ	T cells, NK cells	Activates macrophages, enhances antigen presentation, and promotes the Th1 immune response.

IFN-γ, interferon-gamma; IL, interleukins; TNF-α, tumour necrosis factor-alpha.

**Table 2:** Anti-inflammatory cytokines

Cytokine	Source	Function during malaria clearance
IL-10	T cells, regulatory T cells	Suppresses pro-inflammatory responses, limits tissue damage, and regulates immune homoeostasis.
TGF-β	Various immune cells	Modulates inflammation, promotes regulatory T-cell function, and contributes to tissue repair.
IL-4	T cells, mast cells	Promotes the Th2 immune response, enhancing antibody production and regulating inflammation.
IL-13	T cells, mast cells	Similar to IL-4, involved in Th2 immune responses and anti-inflammatory processes.

IL, interleukins; TGF-β, transforming growth factor-beta.

**Table 3:** Chemotactic cytokines (chemokines)

Cytokine	Source	Function during malaria clearance
CXCL8 (IL-8)	Macrophages, neutrophils	Attracts neutrophils and other immune cells to the site of infection, promoting inflammation.
CCL2 (MCP-1)	Macrophages, monocytes	Recruits monocytes and macrophages to the infected area, facilitating immune cell clearance.
CXCL10 (IP-10)	Various immune cells	Induces migration of T cells, NK cells, and macrophages to enhance the immune response.
CCL22	Dendritic cells, macrophages	Attracts regulatory T cells, playing a role in immune modulation.

IL, interleukins; IP-10, interferon γ-induced protein 10 kDa; MCP-1/CCL2, monocyte chemoattractant protein-1; NK, natural killing.

**Table 4:** Th17-related cytokines

Cytokine	Source	Function during malaria clearance
IL-17A	T cells, neutrophils	Promotes inflammation and recruits immune cells to the infection site.
IL-22	T cells, NK cells	Supports tissue repair and regeneration during the immune response.
IL-23	Macrophages, dendritic cells	Sustains Th17 cell differentiation and promotes the production of IL-17.
IL-21	T cells, NK cells	Regulates Th17 cell differentiation and supports anti-malarial immunity.

IL, interleukins. NK, natural killing.

## Mechanisms by which cytokines contribute to malaria clearance

Cytokines play a crucial role in the immune response against malaria, contributing to both the clearance of the parasite and the resolution of infection^4^. Here are some mechanisms by which cytokines contribute to malaria clearance:

## Activation of immune cells

Pro-inflammatory cytokines such as TNF-α and IFN-γ activate macrophages, enhancing their phagocytic activity. Activated macrophages play a key role in engulfing and destroying malaria parasites.

## Promotion of inflammation

Pro-inflammatory cytokines, including IL-1β and IL-6, stimulate inflammation. This inflammatory response helps recruit immune cells to the site of infection, creating an environment that is hostile to the malaria parasite^4^.

## Enhanced antigen presentation

IFN-γ, among other cytokines, boosts the ability of antigen-presenting cells (APCs) to present malaria antigens to T cells. This activation is crucial for the initiation of an adaptive immune response.

## Th1 and Th2 polarization

Cytokines such as IFN-γ promote a Th1 response, which is associated with cellular immunity and effective clearance of intracellular pathogens. Conversely, IL-4 and IL-13 drive a Th2 response, supporting B-cell activation and antibody production.

## Regulation of immune homoeostasis

Anti-inflammatory cytokines like IL-10 and TGF-β help regulate the immune response, preventing excessive inflammation and tissue damage. IL-10, for instance, can suppress the production of pro-inflammatory cytokines.

## Chemotaxis and recruitment

Chemotactic cytokines (chemokines), including CXCL8 and CCL2, attract immune cells, such as neutrophils and monocytes, to the site of infection. This facilitates the clearance of infected cells and the removal of parasites.

## Support for regulatory T cells

IL-10 and TGF-β contribute to the generation and maintenance of regulatory T cells (Tregs), which help suppress excessive immune responses and maintain immune homoeostasis during malaria infection.

## Th17-mediated responses

Th17-related cytokines like IL-17A and IL-22 can contribute to the immune response against malaria by promoting inflammation, enhancing barrier defenses, and supporting tissue repair.

## Enhancement of antibody responses

Cytokines like IL-4 and IL-21 support B-cell activation and antibody production. Antibodies play a crucial role in preventing malaria parasites from infecting new cells.

## Induction of apoptosis

Cytokines can contribute to the induction of apoptosis in infected cells, facilitating the elimination of malaria parasites.

## Factors that influence the balance of pro-inflammatory and anti-inflammatory cytokines during malaria infection

The balance between pro-inflammatory and anti-inflammatory cytokines during malaria infection is influenced by various factors^57^. The intricate interplay of these factors determines the overall immune response to the parasite. Here are several key factors that influence the balance:

## Parasite strain and load

The strain of the malaria parasite and the overall parasite load can impact the cytokine balance. Higher parasite loads may induce a more pronounced pro-inflammatory response.

## Host genetic factors

Individual genetic variations can influence the host’s immune response. Certain genetic factors may predispose individuals to either a strong pro-inflammatory or anti-inflammatory response during malaria infection^4^.

## Immune status of the host

The host’s immune status, including pre-existing immunity and a history of previous malaria infections, can affect the balance of cytokines. Immune individuals may exhibit a more balanced response.

## Timing and stage of infection

The progression of the infection and the life cycle stage of the malaria parasite (e.g. liver stage, blood stage) can influence the cytokine balance. Different stages may trigger distinct immune responses.

## Co-infections and co-morbidities

Concurrent infections or underlying health conditions can modulate the immune response to malaria. Co-infections may exacerbate inflammation, while certain co-morbidities could skew the response towards an anti-inflammatory profile.

## Nutritional status

Adequate nutrition is essential for a well-functioning immune system. Malnutrition or specific nutrient deficiencies may impact the cytokine balance and the overall immune response during malaria infection.

## Age

The age of the individual can affect the immune response. Children and infants may exhibit different cytokine profiles compared to adults, influencing the severity of the infection and the balance between pro-inflammatory and anti-inflammatory responses.

## Sex hormones

Hormonal differences between males and females can influence the immune response. Studies suggest that sex hormones may impact the balance of pro-inflammatory and anti-inflammatory cytokines during malaria infection.

## Environmental factors

Environmental factors such as temperature, humidity, and exposure to other pathogens can contribute to the complexity of the immune response and the balance between pro- and anti-inflammatory cytokines.

## Immunomodulatory molecules

The presence of immunomodulatory molecules, including cytokines such as IL-10 and TGF-β, plays a direct role in regulating the balance between pro- and anti-inflammatory responses.

## Host immune cell activity

The activity of immune cells, particularly regulatory T cells and macrophages, can influence the cytokine balance. These cells produce both pro- and anti-inflammatory cytokines, contributing to the overall immune milieu.

## Cytokine profile differences between individuals with different clinical manifestations of malaria, such as uncomplicated versus severe disease

The cytokine profiles in individuals with different clinical manifestations of malaria, specifically uncomplicated versus severe disease, vary significantly. The immune response to the malaria parasite involves a complex interplay of pro-inflammatory and anti-inflammatory cytokines^56^.

## Cytokine profiles in uncomplicated malaria

Elevated levels of pro-inflammatory cytokines such as TNF-α, IL-1β, and IFN-γ are typically observed in uncomplicated malaria. These cytokines contribute to the activation of immune cells, enhanced phagocytosis, and the initiation of adaptive immune responses. Increased levels of chemokines like CXCL8 and CCL2 promote the recruitment of immune cells to the site of infection, aiding in parasite clearance. Moderate levels of Th1 cytokines, such as IL-12, support the development of a cellular immune response. A balanced Th1/Th2 response is often present, with IL-10 acting to regulate excessive inflammation and limit tissue damage. IL-4 and IL-13 contribute to the development of anti-malarial antibodies^55^.

## Cytokine profiles in severe malaria

Severe malaria is characterized by an overwhelming pro-inflammatory response, leading to systemic inflammation and tissue damage. Markedly elevated levels of TNF-α, IL-1β, and IL-6 contribute to the severity of the disease. There may be an imbalance in Th1/Th2 responses, with a shift towards excessive Th1 responses. Elevated levels of IFN-γ can contribute to hyperinflammation and immunopathology. While IL-10 is an anti-inflammatory cytokine, in severe malaria, its regulatory function may be impaired or overwhelmed by the excessive pro-inflammatory response. TGF-β may be elevated but may not effectively counterbalance the inflammatory cascade. Dysregulated levels of chemokines, such as excessive CXCL8, may contribute to the recruitment and activation of immune cells, contributing to tissue damage. Severe malaria is associated with increased levels of circulating biomarkers such as soluble TNF receptors and other indicators of systemic inflammation. Severe malaria can lead to immune dysregulation and immunosuppression, affecting the overall cytokine balance. Reduced levels of some cytokines involved in adaptive immune responses may compromise the host’s ability to control the infection^57^.

## Specific cytokine signatures associated with malaria severity and treatment response

The cytokine signatures associated with malaria severity and treatment response can provide valuable insights into the immunopathogenesis of the disease and the effectiveness of therapeutic interventions.

## Cytokine signatures associated with malaria severity

Elevated levels of TNF-α, IL-1β, and IL-6 are commonly associated with severe malaria. These cytokines contribute to systemic inflammation and may be indicative of immune dysregulation. Excessive production of IFN-γ and dysregulated Th1 responses can contribute to severe malaria pathology, including immunopathology and tissue damage. Increased levels of IL-12 and IL-18, which play roles in promoting Th1 responses, are often observed in severe malaria cases. Severe malaria may be characterized by an imbalance in Th1/Th2 responses, with a predominant Th1 response contributing to inflammation and disease severity. While IL-10 is an anti-inflammatory cytokine, its dysregulation, including insufficient regulation of pro-inflammatory responses, may be associated with severe malaria. Increased levels of chemokines, such as CXCL8 and CCL2, may contribute to the recruitment and activation of immune cells, potentially leading to tissue damage. Circulating biomarkers like C-reactive protein (CRP) and soluble TNF receptors may be elevated in severe malaria cases, indicating systemic inflammation^4^.

## Cytokine signatures associated with treatment response

Effective treatment is often associated with a reduction in pro-inflammatory cytokines such as TNF-α, IL-1β, and IL-6, reflecting a decrease in the inflammatory response. Successful treatment leads to a restoration of the Th1/Th2 balance, with a decline in excessive Th1 responses and a return to a more regulated immune profile. Effective treatment helps regulate IFN-γ levels, preventing excessive inflammation while maintaining the immune response against the parasite. Treatment aims to restore the balance of anti-inflammatory cytokines, particularly IL-10, to help regulate the immune response without compromising the ability to clear the infection. Successful treatment results in a decrease in circulating biomarkers of systemic inflammation, such as CRP and soluble TNF receptors. Treatment leads to the normalization of chemokine levels, reducing the recruitment and activation of immune cells and minimizing potential tissue damage. Effective treatment contributes to the restoration of immune homoeostasis, ensuring an appropriate balance between pro- and anti-inflammatory responses^57^.

## Immunomodulation in malaria

Malaria, caused by Plasmodium parasites, presents a complex interaction between the pathogen and the host’s immune system. The parasites have evolved sophisticated mechanisms to manipulate the immune response, allowing them to evade clearance and persist within the host^49^. During the initial stages of infection, Plasmodium parasites encounter the host’s immune cells, triggering a cascade of immune responses. The innate immune system, comprising macrophages, dendritic cells, and natural killer cells, is activated to combat the parasites. In response, the parasites employ various strategies to evade detection and destruction. They modulate the host’s immune responses through several mechanisms, including antigenic variation, sequestration, and manipulation of cytokine profiles^58,59^.

Antigenic variation, a hallmark of Plasmodium parasites, involves altering the surface proteins expressed on infected red blood cells. This continuous change in surface antigens evades the host’s immune recognition, allowing the parasites to evade clearance and persist in the bloodstream^60^. Additionally, *Plasmodium species*, particularly *Plasmodium falciparum,* have the ability to sequester in deep tissues, avoiding detection and destruction by the spleen’s filtering mechanisms. This sequestration in vital organs contributes to disease severity and complicates treatment efforts. Moreover, the parasites can modulate the production and balance of cytokines in the host. They induce both pro-inflammatory and anti-inflammatory cytokines, influencing the immune response’s polarization. This modulation of cytokine profiles affects the severity of symptoms and the development of immunity^61,62^. Furthermore, malaria parasites can impair the function of immune cells, such as T cells and antigen-presenting cells, compromising the host’s ability to mount an effective immune response. This immune dysregulation contributes to the persistence of the infection and the recurrence of malaria episodes^63,64^.

Understanding the mechanisms of immunomodulation by the malaria parasite is crucial for developing effective strategies to combat the disease. Targeting these mechanisms could potentially enhance vaccine development, therapeutic interventions, and the design of anti-malarial drugs. Immunomodulation in malaria represents a sophisticated interplay between the parasite and the host’s immune system. The parasite’s ability to manipulate immune responses allows it to evade clearance mechanisms, leading to the persistence of infection and contributing to the complexity of malaria pathogenesis^65^.

## Cytokine responses to *Plasmodium falciparum*

Upon infection with *Plasmodium falciparum*, the host’s immune system responds by releasing a variety of cytokines, which are signalling molecules that regulate immune responses. The cytokine profile elicited during malaria plays a crucial role in shaping the immune response, controlling parasite growth, and determining disease outcomes^66^. During the early stages of infection, pro-inflammatory cytokines such as IL-12, IFN-γ, and TNF-α are released. These cytokines are essential for activating macrophages and natural killer cells, promoting the initial immune response against the parasites^67^. Conversely, the host also produces regulatory cytokines, notably IL-10 and TGF-β, which act to modulate the immune response. IL-10 and TGF-β help regulate excessive inflammation, preventing immune-mediated tissue damage and contributing to the maintenance of immune homoeostasis^68^.

*Plasmodium falciparum* infection induces a complex interplay between pro-inflammatory and anti-inflammatory cytokines. The balance between these cytokines is crucial for controlling parasite growth while limiting immunopathology. However, an imbalance in cytokine production can lead to immune hyperactivation and contribute to the severity of malaria symptoms^69^. In severe cases of malaria caused by *Plasmodium falciparum,* an excessive and dysregulated immune response can lead to systemic inflammation, endothelial dysfunction, and multi-organ damage. Elevated levels of pro-inflammatory cytokines are often associated with the severity of the disease and can contribute to complications such as cerebral malaria or severe anaemia^34^. The specific cytokine profile induced by *Plasmodium falciparum* infection can vary depending on factors such as host immunity, parasite strain, and disease severity. Understanding these variations in cytokine responses is crucial for elucidating disease mechanisms and developing targeted interventions, including vaccines and immunomodulatory therapies^70^. *Plasmodium falciparum* infection triggers a complex cytokine response characterized by a balance between pro-inflammatory and anti-inflammatory cytokines. The modulation of these cytokines influences the immune response’s effectiveness in controlling parasite growth and determines the severity of malaria, highlighting the importance of cytokine dynamics in malaria pathogenesis^32^.

## Cytokines and chemokines in malaria

Cytokines and chemokines are vital mediators that orchestrate the immune response during malaria infection. Pro-inflammatory cytokines like IL-12, IFN-γ, and TNF-α are crucial in activating the immune system’s initial response against the parasites^71^. They play roles in activating macrophages, natural killer cells, and T cells to combat the infection. Regulatory cytokines such as IL-10 and TGF-β act to modulate and regulate the immune response, preventing excessive inflammation and immune-mediated damage while contributing to immune homoeostasis. The balance between pro-inflammatory and regulatory cytokines is critical in malaria pathogenesis. Imbalances can lead to immune dysregulation, contributing to disease severity and complications^72^.

Chemokines are small signalling proteins responsible for directing the migration and positioning of immune cells within tissues. They play pivotal roles in attracting immune cells to the sites of infection and inflammation during malaria. Chemokines facilitate the recruitment of leucocytes, including monocytes, neutrophils, and lymphocytes, to the sites of Plasmodium infection. Specific chemokines like CXCL8 (IL-8), CCL2 (MCP-1), and CXCL10 (IP-10) are involved in the recruitment of immune cells to the sites of malaria infection. The expression and regulation of chemokines are tightly controlled and contribute significantly to immune cell trafficking and localization during different stages of malaria infection^73–75^.

Understanding the intricate interplay between cytokines and chemokines in malaria is essential for deciphering disease mechanisms, determining immune responses, and developing interventions such as vaccines or immunomodulatory therapies. Targeting specific cytokine or chemokine pathways may offer potential strategies for modulating the immune response and improving treatment outcomes in malaria^76^. Cytokines and chemokines serve as crucial mediators in orchestrating the immune response during malaria infection. Their roles in immune cell activation, regulation, and migration are integral to both host defense and the pathology associated with malaria^77^.

## Outcomes and challenges to potential therapeutic interventions targeting cytokine pathways that have been explored in preclinical and clinical studies

Therapeutic interventions targeting cytokine pathways in malaria have been explored in both preclinical and clinical studies. While some promising outcomes have been observed, several challenges persist.

## Outcomes from preclinical and clinical studies

In preclinical studies, anti-TNF-α therapies showed promise in mitigating severe malaria symptoms and reducing mortality. Clinical studies, however, yielded mixed results, with concerns about increased parasite burden and adverse effects. The dual role of TNF-α in malaria, both protective and pathogenic, poses challenges. Timing and dosage of anti-TNF-α interventions need careful consideration. Preclinical studies targeting IL-1β have demonstrated reduced inflammation and improved survival in severe malaria models. Clinical trials are ongoing, showing potential for therapeutic benefits. Limited data from clinical trials and potential side effects underscore the need for further investigation to establish safety and efficacy. Strategies to modulate IL-10 levels have shown promise in preclinical studies, aiming to balance immune responses and prevent excessive inflammation. However, translation to clinical success remains a challenge. Achieving the right balance of IL-10 modulation is critical, as both insufficient and excessive IL-10 levels may have negative consequences on the immune response. Modulating Th17-related cytokines has shown potential in preclinical studies for regulating inflammation and tissue repair. Clinical trials are limited. The complex role of Th17 responses in malaria and potential impacts on other immune processes necessitates careful examination before widespread therapeutic use. TGF-β plays a role in immune regulation and tissue repair. Preclinical studies exploring TGF-β modulation have shown mixed outcomes. The dual role of TGF-β in both promoting and limiting inflammation poses challenges, requiring precise modulation for therapeutic benefit^74^.

## Common challenges

Precise timing and dosage of interventions are critical. Administering therapies at the right phase of infection and determining optimal dosages for safety and efficacy remain challenging. Genetic and immunological differences among individuals impact responses to cytokine-targeted therapies. Personalized approaches may be necessary. Interconnected cytokine networks make it challenging to modulate a single pathway without affecting others. A comprehensive understanding of cytokine interactions is required. Modulating cytokines may inadvertently contribute to immunopathology. Striking a balance between controlling the infection and preventing excessive inflammation is crucial. Potential adverse effects, including increased susceptibility to other infections or dysregulation of immune responses, need to be carefully monitored. Limited data from clinical trials pose challenges in establishing the safety and efficacy of cytokine-targeted therapies. More robust clinical evidence is necessary^75^.

## Future directions

Future directions in malaria research and therapeutic development involve addressing existing challenges, exploring innovative approaches, and enhancing our understanding of the complex interactions between the parasite and the host immune system. Investigate the impact of host genetics on malaria susceptibility and treatment responses. Develop personalized treatment approaches based on individual genetic profiles to optimize therapeutic interventions. Explore targeted immunomodulation strategies to balance pro-inflammatory and anti-inflammatory responses. Investigate the potential of cytokine-based therapies with careful consideration of timing, dosage, and individual variations. Continue efforts to develop an effective malaria vaccine, considering the complexity of the Plasmodium life cycle. Explore multi-stage and multi-antigen vaccine candidates to enhance protective immunity. Implement robust surveillance programs to monitor and respond to emerging drug resistance. Explore new drug combinations and alternative treatment regimens to combat resistance. Investigate novel therapeutic approaches, including host-directed therapies and adjunctive treatments, to enhance the efficacy of existing anti-malarial drugs. Explore the potential of nanotechnology and other innovative drug delivery systems for improved treatment outcomes. Identify and validate biomarkers for early diagnosis, disease severity prediction, and treatment response monitoring. Develop point-of-care diagnostic tools for rapid and accurate malaria detection in resource-limited settings^76^.

Develop and implement innovative vector control strategies to complement existing interventions. Explore genetic and environmental approaches for sustainable vector control, such as gene-drive technologies. Enhance community engagement and education to improve malaria prevention and treatment practices. Implement targeted health communication strategies to increase awareness and reduce the stigma associated with malaria. Adopt a One Health approach to understand the ecological and environmental factors influencing malaria transmission. Collaborate across disciplines to address the complex interactions between humans, animals, and the environment in malaria-endemic regions. Strengthen international collaboration to share knowledge, resources, and expertise in the fight against malaria. Encourage partnerships between research institutions, governments, NGOs, and the private sector for a comprehensive and coordinated approach. Utilize advanced data analytics and integration methods to analyze large datasets and gain insights into malaria epidemiology, drug efficacy, and host-pathogen interactions. Foster open data sharing and collaboration to accelerate progress in malaria research^74^.

## Conclusion

The role of cytokines in immunomodulation during malaria clearance is intricate and multi-faceted. Cytokines, as signalling molecules, orchestrate the immune response, impacting both the host’s defense mechanisms and the parasite’s survival strategies. During malaria infection, pro-inflammatory cytokines such as IL-12, IFN-γ, and TNF-α contribute to the activation of macrophages and other immune cells. These cytokines stimulate the clearance of the parasite by enhancing phagocytosis and promoting the killing of infected cells. Conversely, excessive or dysregulated production of pro-inflammatory cytokines can lead to immunopathology and severe disease manifestations. High levels of TNF-α and other cytokines can contribute to tissue damage, endothelial dysfunction, and severe complications like cerebral malaria.

Furthermore, anti-inflammatory cytokines such as IL-10 and TGF-β play a crucial role in regulating the immune response, preventing excessive inflammation, and limiting tissue damage. However, their overproduction might also impair parasite clearance by dampening the immune system’s ability to combat the infection effectively. The balance between pro-inflammatory and anti-inflammatory cytokines is crucial for effective malaria clearance while avoiding excessive immunopathology. Understanding this delicate balance is vital for the development of novel therapeutic strategies aiming to modulate the immune response during malaria infection. Cytokines serve as key mediators in regulating the immune response against malaria. Their intricate interplay influences the outcome of infection, affecting both parasite clearance and the severity of the disease. Achieving a balanced cytokine response is pivotal for effective malaria clearance without causing excessive immunopathology, paving the way for potential targeted immunomodulatory therapies to improve treatment outcomes for malaria.

## Ethical approval

Not applicable.

## Consent

Not applicable.

## Sources of funding

No funding was received for writing this review paper.

## Author contribution

E.I.O. performed the following roles: conceptualisation, methodology, supervision, draft writing, editing and approval before submission.

## Conflicts of interest disclosure

The author declares no conflicts of interest.

## Research registration unique Identifying number (UIN)

Not applicable.

## Guarantor

Not applicable as this is a review. It does not have any data.

## Data availability statement

Not applicable.

## Provenance and peer review

Not applicable.
